# Vaccination in Oncology Patients: Evidence, Clinical Challenges, and Future Directions

**DOI:** 10.3390/vaccines14030250

**Published:** 2026-03-09

**Authors:** Alberto Giuseppe Agostara, Silvia Della Torre, Sara Di Bella, Michela Pelliccione, Paola Candido, Valeria Smiroldo, Laura Roazzi, Gabriele Ciarlo, Davide Toniolo, Francesca Zannier, Roberto Bollina

**Affiliations:** 1 Department of Oncology, Ospedale G. Salvini—Rho—ASST Rhodense, Rho, 20017 Milan, Italy; 2 Department of Oncology and Hemato-Oncology, Grande Ospedale Metropolitano Niguarda, 20162 Milan, Italy

**Keywords:** cancer, oncology, vaccination, immunocompromised host, immunogenicity, preventive oncology

## Abstract

This narrative review provides a comprehensive synthesis of patients with cancer disproportionately affected by vaccine-preventable infectious diseases due to malignancy-related immune dysfunction and treatment-induced immunosuppression. Vaccination represents a cornerstone of supportive care in oncology; however, vaccine uptake remains suboptimal and immune responses are frequently attenuated, particularly in patients with hematological malignancies and those receiving highly immunosuppressive therapies. This review provides a comprehensive synthesis of current evidence on vaccine immunogenicity, clinical effectiveness, and safety in oncology patients. We discuss immunological mechanisms underlying impaired vaccine responses, summarize evidence for major vaccines recommended in oncology practice, and address key clinical challenges such as timing relative to anticancer therapies, heterogeneity of immune responses, and vaccine hesitancy. An extended discussion highlights emerging strategies, including booster dosing, adjuvanted vaccines, and personalized vaccination approaches. Finally, future perspectives are outlined to improve integration of vaccination into routine oncology care.

## 1. Introduction

Infectious diseases remain a leading cause of morbidity and mortality among patients with cancer, despite major advances in anticancer therapy and supportive care over recent decades [1,2]. The vulnerability of oncology patients to infectious complications stems from a complex interplay between the underlying malignancy itself and the immunosuppressive effects of modern anticancer treatments, including chemotherapy, radiotherapy, targeted molecular therapies, corticosteroids, and hematopoietic stem cell transplantation (HSCT) [1,2]. This heightened susceptibility translates into substantial clinical consequences, with infections responsible for approximately 50% of deaths in patients with hematological malignancies, particularly in high-income countries, including the United States and Western Europe [1,2], and contributing significantly to mortality in solid tumor populations.

Beyond direct mortality, infectious complications lead to prolonged hospitalizations, treatment delays or interruptions that may compromise cancer outcomes, dose reductions in anticancer therapy, increased healthcare costs running into billions of dollars annually, and significantly diminished quality of life. The economic and social burden extends to patients’ families and healthcare systems, with infection-related hospitalizations representing a major source of unplanned healthcare utilization.

Vaccine-preventable diseases (VPDs) represent a particularly important and potentially modifiable subset of infectious threats in the oncology population. Conditions such as influenza, invasive pneumococcal disease, herpes zoster, hepatitis B virus reactivation, and SARS-CoV-2 infection are all associated with markedly increased hospitalization rates, treatment disruptions, and excess mortality in cancer patients compared to the general population [3,4,5]. For instance, influenza-related mortality in cancer patients can be up to 10 times higher than in age-matched healthy controls, with case-fatality rates reaching 5–10% in certain patient subgroups. Similarly, herpes zoster occurs at rates 3–12 times higher in immunocompromised cancer patients than in immunocompetent individuals, and invasive pneumococcal disease incidence is elevated 5–10-fold in patients with hematological malignancies.

Given this substantial disease burden, vaccination is increasingly recognized as a fundamental and essential component of comprehensive preventive oncology care. International oncology societies, including the American Society of Clinical Oncology (ASCO), the European Society for Medical Oncology (ESMO), and the Infectious Diseases Society of America (IDSA), have all published guidelines strongly recommending routine vaccination for oncology patients [3,4].

Nevertheless, despite this strong professional consensus and clear guideline support, significant barriers continue to limit the consistent implementation of vaccination strategies in clinical oncology practice [5]. These barriers operate at multiple levels: patient-level concerns about vaccine safety and efficacy in immunocompromised states, provider-level uncertainty about optimal timing and which vaccines to prioritize, and health system-level logistical challenges, including lack of on-site vaccination services and unclear responsibility between oncology and primary care physicians.

Vaccination coverage rates remain disappointingly low across multiple studies and healthcare systems in North America and Europe. Only 30–60% of eligible cancer patients receive recommended influenza vaccination annually, rates for pneumococcal vaccination are even lower at 20–40%, and uptake of newer vaccines such as recombinant zoster vaccine is often below 10% in oncology populations [6,7]. These coverage rates fall far short of public health targets and represent major missed opportunities for prevention of serious infectious complications.

The COVID-19 pandemic brought renewed attention and urgency to vaccination in immunocompromised populations, including cancer patients [8,9,10]. Additionally, the extensive research conducted on SARS-CoV-2 vaccine immunogenicity in cancer patients has provided valuable insights into the heterogeneity of vaccine responses across different cancer types and treatment modalities, lessons that extend beyond COVID-19 to inform our broader approach to vaccination in oncology.

## 2. Immunological Basis of Vaccine Responses in Oncology Patients

### 2.1. Cancer-Associated Immune Dysfunction

Malignancies themselves induce profound alterations in both innate and adaptive immunity even before any therapeutic intervention, creating a state of chronic immunosuppression that compromises vaccine responses [11]. The mechanisms of cancer-associated immune dysfunction are complex, multifaceted, and often interdependent, affecting multiple levels of the immune system simultaneously.

Chronic inflammation represents one of the hallmarks of cancer and contributes to immune dysfunction through multiple pathways [11]. The tumor microenvironment is characterized by elevated levels of pro-inflammatory cytokines, including interleukin-6 (IL-6), tumor necrosis factor-alpha (TNF-α), and interleukin-1 (IL-1). While acute inflammation is generally beneficial for host defense and immune activation, chronic inflammation paradoxically impairs effective immunity through several mechanisms. These include immune cell exhaustion from persistent stimulation, metabolic reprogramming that limits immune cell function, and skewing of immune responses toward tolerogenic rather than protective phenotypes.

T cell exhaustion is another cardinal feature of cancer-associated immune dysfunction that has important implications for vaccine responses. Tumor-specific T cells become progressively dysfunctional through sustained exposure to tumor antigens in the absence of adequate co-stimulatory signals, a process characterized by upregulation of multiple inhibitory receptors, including PD-1, CTLA-4, TIM-3, and LAG-3 [11]. Exhausted T cells exhibit reduced proliferative capacity following antigen stimulation. Importantly, this exhaustion phenomenon can extend beyond tumor-specific T cells to affect the broader T cell repertoire through “bystander exhaustion,” potentially compromising responses to vaccine antigens even in patients with localized solid tumors who do not have systemic disease and have not yet received immunosuppressive therapy.

Expansion of immunosuppressive cell populations represents a third major mechanism by which cancer impairs immunity. Tumors actively recruit and expand regulatory T cells (Tregs), myeloid-derived suppressor cells (MDSCs), and tumor-associated macrophages with M2 polarization, all of which contribute to an immunosuppressive milieu [11]. These cells exert their suppressive effects through multiple mechanisms: secretion of inhibitory cytokines, including IL-10 and TGF-β, that dampen immune responses, expression of inhibitory ligands such as PD-L1 and PD-L2 that engage inhibitory receptors on effector T cells, consumption of essential amino acids through enzymes like arginase and indoleamine 2,3-dioxygenase (IDO), thereby limiting T cell proliferation, and production of reactive oxygen and nitrogen species that directly impair T cell function and survival.

In hematological malignancies, the situation is fundamentally different and typically more severe because the cancer arises from cells of the immune system itself [12,13]. Diseases such as chronic lymphocytic leukemia (CLL), multiple myeloma, lymphomas, and acute leukemias directly impair B cell function, T cell function, or both, depending on the lineage of malignant cells.

### 2.2. Impact of Anticancer Therapies

Modern cancer treatment regimens, while highly effective at controlling malignancy, invariably cause varying degrees of immunosuppression that profoundly affect vaccine responses. The nature and duration of treatment-induced immunosuppression vary substantially depending on the specific therapeutic agent, dose intensity, duration of treatment, and individual patient factors.

Cytotoxic chemotherapy induces transient or prolonged lymphopenia, negatively affecting vaccine responsiveness [14]. Different chemotherapy agents vary considerably in their immunosuppressive effects. Alkylating agents such as cyclophosphamide, ifosfamide, and bendamustine cause particularly profound and long-lasting immunosuppression, often resulting in lymphopenia that persists for months to years after treatment completion. Purine analogs including fludarabine and cladribine have similarly severe and prolonged effects on lymphocyte populations. These agents deplete both B cells and T cells, impair lymphocyte proliferation capacity, and may damage lymphoid organs including the thymus, leading to defects in immune reconstitution. Antimetabolites such as methotrexate and 5-fluorouracil cause more transient immunosuppression that generally resolves within 1–2 weeks after each treatment cycle, though cumulative effects can occur with repeated cycles. Platinum compounds including cisplatin and carboplatin have moderate immunosuppressive effects, while anthracyclines affect immune function to a lesser degree than alkylating agents.

The cumulative nature of chemotherapy-induced immunosuppression is particularly important to recognize in clinical practice. This is especially relevant for high-dose chemotherapy regimens used in curative-intent settings such as adjuvant or neoadjuvant chemotherapy for breast cancer, colon cancer, or lung cancer, where patients may receive 4–6 months of intensive treatment. High-dose chemotherapy with autologous stem cell rescue, used for lymphomas and other hematological malignancies, causes profound immunosuppression for several months post-transplant with gradual immune reconstitution occurring over 12–24 months. Allogeneic HSCT causes even more severe and prolonged immunosuppression, with full immune reconstitution sometimes taking 2–3 years or longer, particularly if chronic graft-versus-host disease develops, requiring continued immunosuppressive therapy.

Corticosteroids are frequently used in oncology for various indications [14]. Corticosteroids have broad immunosuppressive effects including induction of lymphopenia through apoptosis of lymphocytes, impaired T cell activation and proliferation, reduced antibody production by B cells, decreased cytokine secretion, and inhibition of antigen presentation by dendritic cells. Even relatively brief courses of moderate to high-dose corticosteroids, such as dexamethasone used for chemotherapy-related nausea, can transiently impair vaccine responses for 1–2 weeks. The immunosuppressive effects are dose-dependent and duration-dependent, with higher doses and longer treatment courses causing more profound impairment.

B cell-depleting monoclonal antibodies, particularly anti-CD20 antibodies including rituximab, obinutuzumab, and ofatumumab used extensively for B cell lymphomas and chronic lymphocytic leukemia, cause profound and prolonged hypogammaglobulinemia and severely impaired antibody responses to vaccination [15]. These agents work by binding to the CD20 antigen expressed on B cells and inducing B cell depletion through multiple mechanisms including complement-dependent cytotoxicity, antibody-dependent cellular cytotoxicity, and direct induction of apoptosis. Following anti-CD20 therapy, CD20-expressing B cells are depleted from the circulation for 6–12 months or longer in most patients, during which time the capacity to mount antibody responses to new antigens including vaccines is markedly impaired or absent. Importantly, even after quantitative B cell recovery with return of normal B cell counts in peripheral blood, functional recovery lags behind, and patients may have qualitatively defective antibody responses for extended periods beyond numerical B cell reconstitution. Whenever clinically feasible, core vaccines should be completed before initiation of B cell-depleting therapy to maximize serological responses.

Studies of influenza, pneumococcal, and COVID-19 vaccination in patients receiving anti-CD20 therapy have consistently demonstrated seroconversion rates of 0–30%, markedly lower than the 70–90% rates in patients not receiving B cell-depleting therapy [15,16]. The profound impairment of humoral responses means that vaccination within 6 months after rituximab or related agents is associated with very poor serological outcomes. The optimal timing for vaccination to maximize responses is at least 6–12 months after the last dose of anti-CD20 therapy, when B cell reconstitution is more complete. Conversely, vaccination before initiation of anti-CD20 therapy can generate protective antibody responses that may persist during subsequent therapy, highlighting the critical importance of proactive vaccination assessment in newly diagnosed patients before treatment initiation.

Other novel targeted agents also significantly affect immunity, though often in more selective ways compared to traditional chemotherapy. Bruton’s tyrosine kinase (BTK) inhibitors including ibrutinib and acalabrutinib, used for chronic lymphocytic leukemia and B cell lymphomas, impair B cell receptor signaling pathways essential for B cell activation, proliferation, and antibody production, resulting in poor vaccine responses similar to, though generally less severe than, anti-CD20 antibodies [16]. Anti-CD38 monoclonal antibodies such as daratumumab and isatuximab, used for multiple myeloma, deplete both malignant plasma cells and normal plasma cells, compromising antibody production capacity. These agents also deplete natural killer cells which express CD38, potentially affecting cellular immunity.

Immune checkpoint inhibitors including anti-PD-1 antibodies (nivolumab, pembrolizumab, cemiplimab), anti-PD-L1 antibodies (atezolizumab, durvalumab, avelumab), and anti-CTLA-4 antibodies (ipilimumab, tremelimumab) represent a distinct category with more nuanced and complex effects on vaccine responses [17,18]. These agents work by releasing inhibitory signals on T cells, thereby enhancing antitumor immunity through restoration of T cell function. In theory, by “releasing the brakes” on the immune system, checkpoint inhibitors might actually enhance rather than impair vaccine responses. Current evidence suggests that vaccination during immune checkpoint inhibitor therapy is safe and does not significantly increase the risk of immune-related adverse events, which was initially a major theoretical concern among oncologists and patients [17,18]. Multiple large observational studies, systematic reviews, and meta-analyses have now confirmed that influenza vaccination in patients receiving ICIs does not increase the incidence or severity of immune-related adverse events such as pneumonitis, colitis, hepatitis, or endocrinopathies [18,19], as corroborated by a systematic review by Cortellini et al. [20]. In fact, some studies have suggested potential survival benefits associated with influenza vaccination in ICI-treated patients, though this provocative finding requires validation in prospective randomized studies [19].

Regarding vaccine immunogenicity during ICI therapy, results have been somewhat mixed across different studies. Some investigations report normal or near-normal antibody responses to influenza and COVID-19 vaccines in ICI-treated patients, while others show somewhat attenuated responses compared to healthy controls but superior to patients receiving chemotherapy. T cell responses to vaccines appear to be well preserved or even enhanced during ICI therapy. Studies specifically evaluating herpes zoster vaccination during checkpoint inhibitor therapy have demonstrated excellent immunogenicity with robust antibody and T cell responses [20,21]. Overall, the accumulated data strongly support the safety and feasibility of vaccination during ICI therapy, with immunogenicity that is generally acceptable though may vary by vaccine type, timing relative to ICI administration, and underlying tumor type.

Radiotherapy effects on systemic immunity depend on several factors including the anatomical field irradiated, total radiation dose, fractionation schedule, and use of concurrent systemic therapies. Localized radiotherapy to non-lymphoid tissues such as extremity soft tissue sarcomas or localized prostate cancer generally has minimal systemic immunosuppressive effects and does not significantly impair vaccine responses. However, radiation to lymphoid organs including the mediastinum (as in Hodgkin lymphoma), abdomen, or pelvis can cause substantial lymphopenia by directly damaging circulating lymphocytes passing through the irradiated field and by affecting lymphoid tissues. Large-field radiotherapy and higher doses are associated with more profound lymphopenia.

### 2.3. Correlates of Protection

A fundamental challenge in vaccinating immunocompromised cancer patients is determining what constitutes protective immunity in this population [21,22]. In immunocompetent individuals, well-established serological correlates of protection exist for many vaccines based on decades of research including controlled challenge studies and large epidemiological analyses. For example, hemagglutination inhibition (HAI) titers ≥1:40 for influenza vaccines, anti-pneumococcal capsular polysaccharide IgG concentrations ≥0.35 µg/mL for pneumococcal vaccines, and anti-spike IgG titers above specific thresholds for SARS-CoV-2 vaccines are generally accepted as correlating with clinical protection in healthy populations.

However, the applicability and validity of these conventional serological thresholds established in immunocompetent populations to immunocompromised cancer patients is highly questionable for several important reasons. Multiple factors influence antibody quality beyond simple quantification, including antibody avidity (the strength of antibody–antigen binding, with higher avidity generally correlating with better protection), epitope specificity and breadth (antibodies targeting multiple epitopes or conserved regions may provide broader protection), isotype and subclass distribution (IgG1 and IgG3 subclasses generally have superior effector functions compared to IgG2 and IgG4), and effector functions such as complement fixation, antibody-dependent cellular cytotoxicity, and viral neutralization capacity [22,23,24,25,26,27].

Second, the kinetics and durability of antibody responses differ in immunosuppressed individuals, with more rapid waning commonly observed. A cancer patient might achieve protective antibody levels at 1 month post-vaccination but fall below protective thresholds by 3–6 months, whereas a healthy individual might maintain protective levels for a full year or longer. This has important implications for booster vaccination strategies and the need for more frequent revaccination in cancer populations [28,29,30,31,32].

Third, and perhaps most importantly, cellular immune responses—particularly T cell immunity—may play a compensatory and critical role when humoral responses are impaired or absent [22,23]. T cell immunity encompasses both CD4+ helper T cells, which support B cell responses through provision of help signals and cytokines but also exert direct antiviral and antibacterial effects, and CD8+ cytotoxic T cells, which recognize and eliminate infected cells displaying viral or bacterial antigens on their surface. The critical importance of T cell immunity in immunocompromised hosts has been clearly and repeatedly demonstrated in studies of SARS-CoV-2 vaccination in cancer patients, particularly those with hematological malignancies receiving B cell-depleting therapies [22,23].

The practical clinical implication of this evolving understanding is that sole reliance on antibody titer measurements to assess vaccine “success” or “failure” may substantially underestimate the true protective benefit of vaccination in cancer patients. A patient with undetectable antibodies following vaccination might be considered a “non-responder” based on serological testing alone, yet that same patient might have mounted a strong T cell response providing significant protection. Conversely, absence of seroconversion does not necessarily equate to vaccination failure or lack of benefit—cellular immunity may still confer meaningful protection even in the absence of detectable antibodies. This recognition has led to increased interest in measuring T cell responses following vaccination in immunocompromised patients to gain a more complete picture of vaccine-induced immunity [22,23].

Another important consideration often overlooked in discussions of vaccine responses is the potential for immunological memory even in the absence of high circulating antibody levels. Immunological memory consists of long-lived memory B cells capable of rapidly differentiating into antibody-secreting plasma cells upon antigen re-exposure, and memory T cells that can quickly proliferate and execute effector functions when encountering their cognate antigen. A cancer patient might have low or even undetectable antibody levels months after vaccination (due to waning of short-lived plasma cells) yet retain robust memory B and T cell populations capable of mounting rapid anamnestic responses upon pathogen exposure. Measuring memory responses requires specialized assays and is not part of routine clinical practice but represents an important dimension of vaccine-induced immunity [16].

From a clinical perspective, the uncertainty and complexity regarding correlates of protection in immunocompromised hosts has several practical consequences that should guide clinical practice. First, it provides strong scientific rationale for proceeding with vaccination even when suboptimal serological responses are anticipated, as some immunity—whether humoral, cellular, or memory-based—is unequivocally better than no immunity. Second, it suggests that booster vaccination strategies employing additional doses may be particularly beneficial to both enhance initial responses and sustain protective immunity over time. Third, it underscores the importance of layered protection strategies that complement vaccination, including vaccination of household contacts and healthcare workers (the “cocooning” strategy to create a protected environment around vulnerable patients), continued attention to infection prevention measures such as hand hygiene and mask use during high-risk periods like influenza season, appropriate use of antimicrobial prophylaxis when indicated based on specific clinical scenarios, and early treatment of infections when they do occur.

## 3. Evidence for Major Vaccines in Oncology Patients

This section reviews the evidence for six vaccine categories that are most relevant to oncology practice and are included in major international guidelines (ASCO, ESMO, IDSA): influenza, pneumococcal, SARS-CoV-2, herpes zoster, hepatitis B, and HPV vaccines. These vaccines were selected based on (1) the markedly increased risk of the corresponding infections in cancer patients compared to the general population, (2) the availability of clinical data specifically in oncology cohorts, and (3) their inclusion in current guideline recommendations for immunocompromised patients [3,4].

### 3.1. Influenza Vaccinations

Seasonal influenza represents one of the most important vaccine-preventable infections in cancer patients, with case-fatality rates of 5–10% in certain subgroups [3,24,25]. Meta-analyses demonstrate that inactivated influenza vaccines achieve seroprotection rates of 52–84% across oncology populations, significantly lower than >90% in healthy adults but still clinically meaningful [24,25]. Solid tumor patients achieve better responses (70–90%) than hematological malignancy patients (40–70%), with poorest responses in those receiving B cell-directed therapies [24,25].

High-dose influenza vaccines (60 µg hemagglutinin per strain) achieve superior immunogenicity compared to standard-dose formulations in immunocompromised adults [26]. ASCO guidelines specifically recommend high-dose vaccine for HSCT recipients [3]. Adjuvanted vaccines containing MF59 or AS01B have shown enhanced responses in cancer patients [27].

Studies comparing vaccination timing relative to chemotherapy found no clinically significant differences between administration on the day of chemotherapy versus 2 weeks prior [28]. Current guidelines recommend vaccination at any opportunity during flu season rather than waiting for optimal timing [3,27]. Safety profiles are excellent with no evidence of increased adverse events, tumor progression, or interference with anticancer therapy [24,25]. Annual revaccination is recommended regardless of prior vaccination history, given ongoing antigenic drift of circulating influenza strains and evidence that repeat vaccination does not reduce immunogenicity in oncology populations.

### 3.2. Pneumococcal Vaccination

Patients with cancer face 5–10-fold higher risk of invasive pneumococcal disease (IPD) compared to healthy individuals, with hematological malignancy patients at particularly elevated risk [28,29]. Pneumococcal conjugate vaccines (PCVs) induce T cell-dependent responses and are more immunogenic than polysaccharide vaccines in immunocompromised patients [29,30]. Studies demonstrate seroprotection rates exceeding 64% for most serotypes [28,29].

Current guidelines recommend sequential vaccination with pneumococcal conjugate vaccine (PCV15, PCV20, or PCV13) followed by 23-valent polysaccharide vaccine (PPSV23) ≥8 weeks later for broader serotype coverage [3,4,30]. The conjugate vaccine primes the immune system, enabling enhanced responses to subsequent polysaccharide vaccination. Real-world studies show pneumococcal vaccination in hematological malignancy patients is associated with 30–50% reductions in pneumonia-related hospitalizations [31].

### 3.3. SARS-CoV-2 Vaccination

COVID-19 vaccines have been extensively evaluated in oncology populations [8,9,10,32]. Patients with solid tumors generally achieve satisfactory responses with seroconversion rates of 85–95% after two mRNA vaccine doses, approaching but not matching the >95% in healthy controls [8,9]. In contrast, hematological malignancy patients exhibit markedly reduced responses of 40–70% depending on disease and treatment [9,10,32]. It should be noted that the large majority of available immunogenicity and safety data in oncology patients derive from studies of mRNA platforms (BNT162b2 and mRNA-1273); extrapolation to vector-based or protein-subunit vaccines should therefore be made with caution.

Patients with chronic lymphocytic leukemia have seroconversion rates of 40–70%, while those receiving anti-CD20 antibodies achieve only 10–40% [10,32]. Multiple myeloma patients show 50–80% responses depending on treatment [33]. Importantly, even when antibody responses are minimal, T cell responses may be preserved, providing some degree of cellular immunity [22,34].

Booster vaccination strategies substantially improve responses. Third doses increased solid tumor seroconversion from 90% to 95–98% and converted 30–50% of hematological malignancy non-responders to responders [35,36]. This evidence supports recommendations for extended primary series (3 doses) plus boosters for immunocompromised patients [4,37].

Safety data confirm no increased adverse events in cancer populations and no evidence of tumor progression or reduced anticancer therapy efficacy [8,9]. In ICI-treated patients, extensive data show no increased immune-related adverse events [17,18,20,21].

### 3.4. Herpes Zoster Vaccination

Herpes zoster risk is 3–12-fold higher in cancer populations, with cumulative incidence of 10–20% over 5 years in hematological malignancies [38,39]. The adjuvanted recombinant zoster vaccine (RZV, Shingrix) has revolutionized prevention, demonstrating 68% efficacy in adults with hematological malignancies receiving immunosuppressive therapy [39]. This remarkable efficacy in profoundly immunosuppressed patients reflects the potent AS01B adjuvant system.

Immunogenicity studies show seroconversion rates exceeding 60% across hematological malignancy subgroups and 80–95% in solid tumor patients [39,40]. Studies in ICI-treated patients demonstrated excellent responses with >90% seroconversion and robust T cell responses [21]. RZV is administered as a two-dose series 2–6 months apart. Both doses are necessary for optimal protection—efficacy after a single dose is only 40–50% versus approximately 70% after completion [41].

RZV is more reactogenic than many vaccines but generally well-tolerated, with local reactions in 60–80% and systemic symptoms in 40–60% of recipients that resolve within 24–48 h [39,40,41]. Guidelines recommend RZV for all adults ≥ 50 years and immunocompromised adults ≥ 19 years [3,4,41].

### 3.5. Hepatitis B and HPV Vaccination

All cancer patients should undergo HBV serological testing before immunosuppressive therapy [42]. HBsAg-positive patients require antiviral prophylaxis. Seronegative patients should ideally be vaccinated before treatment, though responses vary substantially (60–80% in solid tumors, 40–60% during chemotherapy, <30% with B cell-directed therapies). Strategies to enhance responses include higher antigen doses (40 µg), additional doses, and adjuvanted formulations [42]. Post-vaccination anti-HBs titer testing is recommended as standard practice to confirm protective immunity (anti-HBs ≥ 10 mIU/mL) before initiating immunosuppressive therapy, given the variability of responses in this population.

HPV vaccination is relevant for young cancer survivors who have elevated risk of HPV-associated second malignancies [43]. The 9-valent HPV vaccine should be offered to survivors in recommended age groups (9–26 years, with consideration for adults 27–45). Vaccination should ideally occur before or after highly immunosuppressive therapy when immune recovery is complete [43]. It should be noted that robust immunogenicity and effectiveness data for the 9-valent HPV vaccine in adult oncology patients actively receiving anticancer therapy remain limited; current recommendations are largely extrapolated from studies in immunocompetent adults and transplant recipients.

A comprehensive summary of vaccine recommendations, immunogenicity, safety profiles, and special considerations for oncology patients is provided in Table 1.

## 4. Clinical Challenges in Oncology Vaccination

### 4.1. Timing Relative to Cancer Therapy

Uncertainty about optimal timing is frequently cited by oncology providers, often leading to missed opportunities. Large trials comparing vaccination on the day of chemotherapy versus 2 weeks prior found no clinically significant differences for influenza and pneumococcal vaccines [28]. Some studies suggest modestly better responses mid-cycle when lymphocyte counts are recovering, but differences are generally small [28].

The critical insight is that timing concerns should not become barriers—it is better to vaccinate at any opportunity than wait indefinitely for optimal conditions. Practical recommendations include: ideally vaccinate before starting chemotherapy when feasible; for patients already on treatment, vaccinate without waiting for breaks; and continue vaccinations even during intensive chemotherapy, as some immunity is better than none [3,4].

For B cell-depleting antibodies, vaccination before treatment provides the best opportunity for responses. Vaccination within 6 months after anti-CD20 yields poor responses. Optimal timing is ≥6–12 months after the last dose, though vaccination should not be withheld indefinitely in patients on maintenance therapy [15]. For ICIs, vaccinate at any time during therapy without specific timing considerations [17,18,19,20,21].

### 4.2. Vaccine Hesitancy and Organizational Barriers

Vaccine hesitancy among cancer patients stems from multiple sources including safety concerns specific to immunocompromised status (worries that vaccines may be dangerous when immunity is weakened, fears about triggering infections or autoimmune reactions), doubts about efficacy (questioning whether vaccines can work when the immune system is compromised, leading to therapeutic nihilism), information gaps (lack of clear communication from healthcare providers about what vaccines are recommended and why), and misinformation exposure (cancer patients are particularly vulnerable to health misinformation encountered through internet sources, social media, or well-meaning but misinformed friends and family) [45,46]. Additionally, some patients harbor pre-existing vaccine hesitancy rooted in philosophical, religious, or political beliefs that predates their cancer diagnosis and may be amplified by the stress and anxiety of cancer treatment.

Among the most prevalent vaccine-related misconceptions specifically documented in oncology settings are: (i) the belief that inactivated vaccines can directly “cause” infection in immunocompromised patients—a concern that is unfounded for all inactivated, subunit, recombinant, and mRNA vaccines currently recommended in oncology, which contain no live replicating pathogen; (ii) fears that vaccination may accelerate tumor progression or interfere with the mechanism of action of anticancer drugs, for which no supporting evidence exists and which is directly contradicted by the safety data reviewed in Section 3; (iii) the extrapolation that because the immune system is “too weak” vaccination is pointless—a conclusion that ignores the clinically meaningful partial protection conferred by even attenuated responses, as well as the documented role of cellular immunity in the absence of seroconversion; and (iv) amplified distrust of vaccine safety in general, fueled by anti-vaccine narratives circulating on social media platforms, including exaggerated reports of adverse events and unsubstantiated claims about vaccine ingredients, that are not specific to oncology but are disproportionately internalized by patients who are anxious about their health and actively searching for information about their condition [45,46,47].

Cancer patients represent a uniquely information-seeking and emotionally vulnerable population. They are more likely than the general public to consult the internet for health information, including vaccine-related content, and to belong to peer support groups where health misinformation can spread rapidly. Published evidence documents that anti-vaccine content online is emotionally resonant and algorithmically amplified relative to accurate scientific information, creating an asymmetric information environment in which patients may encounter multiple fearful narratives for each reassuring one [46,47]. Oncologists and cancer care teams are therefore encouraged to proactively anticipate common misconceptions during consultations, rather than waiting for patients to raise concerns. Directing patients to trusted written resources—such as ASCO’s Cancer.Net patient information portal, national public health authority vaccine information pages, and peer-reviewed lay summaries—can help counteract misinformation encountered outside the clinical encounter. The importance of reliable communication is further underscored by cross-national survey data showing that drivers of vaccine hesitancy vary by country and cultural context, with trust in healthcare institutions and perceived vaccine safety being the most modifiable factors across diverse healthcare settings [45,46,47].

Healthcare provider recommendation is consistently identified as the strongest predictor of vaccine acceptance across multiple studies in cancer populations [47]. Patients who receive clear, personalized recommendations from their oncologist or members of the cancer care team are far more likely to accept vaccination than those who do not receive such guidance or receive ambiguous messages. Conversely, lack of provider recommendation—whether due to oversight, uncertainty about guidelines, time constraints during visits, or assumption that someone else (primary care physician, public health services) will address it—is a major driver of vaccine refusal or deferral that ultimately translates to missed opportunities. Notably, even patients who have been exposed to anti-vaccine misinformation are substantially more likely to accept vaccination when their oncologist frames it as a direct, personalized, and non-optional recommendation, rather than presenting it as an optional choice [47].

Evidence-based approaches to improve vaccine acceptance include proactive provider recommendations delivered in a presumptive tone (“We recommend flu shots for all our cancer patients” rather than “Would you like to consider getting a flu shot?”), personalized risk–benefit discussions tailored to the individual patient’s cancer type and treatment regimen, explicit and patient-friendly rebuttals of common misconceptions (e.g., clearly explaining that inactivated vaccines cannot cause the diseases they prevent and that vaccination is safe during most chemotherapy regimens), setting realistic expectations about vaccine efficacy while emphasizing that partial protection is valuable, and leveraging trusted messengers throughout the cancer care team including oncology nurses, pharmacists, and patient navigators who may have more time for extended counseling conversations [45,46,47].

Organizational and health system-level barriers substantially contribute to suboptimal vaccination coverage and include lack of systematic assessment of vaccination status during oncology visits, unclear responsibility with ambiguity about whether the oncology team or primary care physician should provide vaccines (often resulting in neither doing so), absence of standing orders requiring individual physician orders for each vaccine rather than allowing nurse-driven protocols, lack of on-site vaccine availability in many oncology clinics necessitating referrals that patients may not complete, and inadequate electronic health record functionality with missing immunization registries, clinical decision support prompts, or automated reminders [44,48]. Additional logistical barriers include workflow disruption concerns, reimbursement uncertainties, staff knowledge gaps, and inventory management challenges. While these barriers have been documented across multiple healthcare systems in North America and Europe [44,48], their relative importance may differ by healthcare model, and country-specific data should be considered when designing targeted interventions.

Successful interventions employ multiple complementary strategies: integration into clinical workflows with vaccination checkpoints at key timepoints, standing order protocols enabling nurses or pharmacists to assess eligibility and administer vaccines, electronic health record enhancements with clinical decision support alerts, on-site vaccine availability, dedicated vaccination programs such as pharmacist-led or nurse-led clinics, education and training for cancer care teams, patient reminders and outreach, performance monitoring with vaccination rates as quality metrics, and multidisciplinary collaboration involving oncology, primary care, infectious diseases, pharmacy, and public health to clarify roles and ensure coordination [49,50]. Additional strategies include the use of patient portals and social media platforms to disseminate evidence-based vaccine information from trusted healthcare providers, thereby countering health misinformation in an accessible format. Furthermore, integrating vaccination rates into cancer center quality metrics and accreditation standards can drive systemic change by creating institutional accountability for vaccination coverage [44,49]. A particularly successful model from an Italian oncology center combining on-site clinics, electronic reminders, nurse-driven protocols, and patient education achieved influenza vaccination rates exceeding 85% and pneumococcal rates of 65%, substantially higher than national averages [50].

## 5. Strategies to Optimize Vaccination Outcomes

### 5.1. Booster Dosing and Extended Primary Series

COVID-19 vaccination experience demonstrated that additional doses substantially improve responses in cancer patients [35,36]. Third mRNA doses converted 30–50% of initial non-responders, and fourth doses provided incremental benefit. This evidence supports extended primary series (3 doses) plus subsequent boosters for immunocompromised patients [4,37]. Similar principles may apply to other vaccines, with booster strategies particularly important in profoundly immunosuppressed populations.

### 5.2. High-Dose and Adjuvanted Formulations

High-dose influenza vaccine with 4-fold higher antigen content demonstrates enhanced antibody responses with 10–25% higher seroprotection rates in immunocompromised populations [26]. ASCO guidelines recommend high-dose vaccine for HSCT recipients [3].

Adjuvanted vaccines employ immunostimulatory substances that enhance responses through innate immune activation. The AS01B adjuvant system (used in RZV) powerfully stimulates both humoral and cellular immunity, enabling high efficacy even in immunosuppressed patients [39,40,41]. MF59-adjuvanted influenza vaccines show enhanced immunogenicity in cancer patients [27]. Novel adjuvants including CpG oligodeoxynucleotides and combination systems are under investigation.

### 5.3. Immune Monitoring and Personalized Approaches

Measurement of vaccine-induced immune responses could enable personalized strategies, identifying patients needing additional doses and guiding booster timing [51,52,53]. However, routine monitoring faces challenges including lack of validated correlates of protection in immunocompromised patients, assay variability, cost and availability concerns, and unclear actionability for many vaccines [51,52]. Despite these challenges, immune monitoring holds particular promise for high-risk subgroups in whom standard vaccination schedules are least likely to be adequate. Patients undergoing B cell-depleting therapy (e.g., anti-CD20 monoclonal antibodies) represent a prime example: given the near-complete abrogation of humoral responses in this population, post-vaccination serological and T cell monitoring could identify true non-responders and guide individualized decisions about additional doses, alternative vaccine platforms, or preventive strategies such as passive immunization with monoclonal antibodies [15,16]. The development of point-of-care assays to assess both antibody and cellular immunity could make such personalized approaches feasible in routine oncology practice.

Current guidelines generally do not recommend routine serological testing except for specific scenarios: hepatitis B post-vaccination testing to confirm protective levels; COVID-19 antibody testing in highest-risk patients (anti-CD20 therapy, recent HSCT) to identify complete non-responders who might benefit from additional interventions [3,4]. As point-of-care assays become available, serological monitoring may expand its role in guiding personalized vaccination.

### 5.4. Household Contact Vaccination and Cocooning Strategy

**Figure 1:**
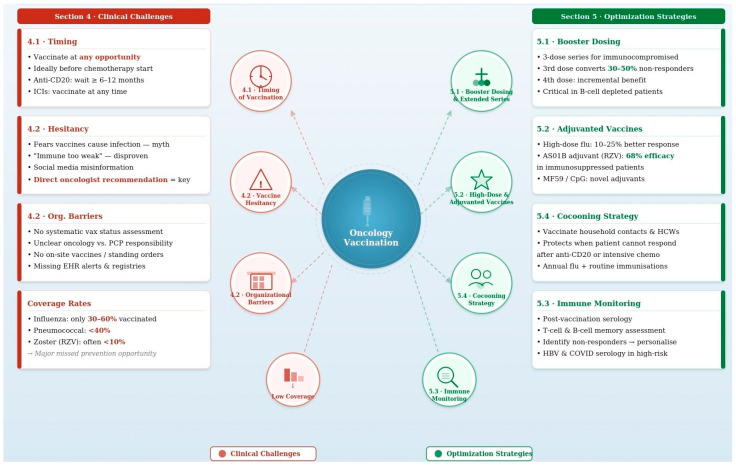
Clinical challenges and optimization strategies in oncology vaccination.

An important but often underemphasized complementary strategy is the vaccination of household contacts and healthcare workers caring for immunocompromised oncology patients—the so-called “cocooning” strategy. When a patient’s own immune response is insufficient to generate protective immunity (e.g., after anti-CD20 therapy or during intensive chemotherapy), creating a protected environment through vaccination of close contacts can substantially reduce the patient’s exposure to vaccine-preventable pathogens, particularly influenza and pertussis. International guidelines recommend that all household members and close contacts of immunocompromised cancer patients receive annual influenza vaccination and be up to date with other routine immunizations [3,4]. Healthcare professionals who regularly interact with oncology patients should similarly be vaccinated, and vaccination status should be actively monitored as part of occupational health programs. This layered approach to protection is especially relevant for patients who cannot mount adequate vaccine responses themselves and represents a direct, actionable strategy that can be implemented alongside patient vaccination programs. A panoramic summary of clinical challenge and optimization strategies in oncology vaccination is provided in Figure 1.

## 6. Discussion and Future Perspectives

The evidence reviewed here supports that vaccination is both safe and clinically beneficial in oncology patients, even when immunogenicity is attenuated. A major lesson from the COVID-19 pandemic is that repeated vaccination and adaptive strategies can substantially improve protection in immunocompromised populations [34,38,39]. It is important to acknowledge, however, that most of this evidence derives from observational studies, retrospective cohort analyses, and post hoc subgroup analyses rather than from randomized controlled trials specifically designed to evaluate vaccination strategies in oncology populations. This evidence gap underscores the need for prospective, randomized vaccine strategy trials in well-defined oncology cohorts.

Heterogeneity of vaccine responses highlights the limitations of uniform recommendations and supports a shift toward personalized approaches. Factors such as cancer type, treatment regimen, lymphocyte counts, and disease stage all influence vaccine immunogenicity and should be considered when developing individualized vaccination plans [35,36]. From a health-system perspective, integrating vaccination into oncology care pathways may reduce infection-related morbidity, prevent treatment interruptions, and improve overall outcomes [5,54,55,56,57].

From an immunological standpoint, future research should focus on identifying reliable correlates of protection and predictive biomarkers to guide individualized vaccination strategies [58]. Novel vaccine platforms, including adjuvanted formulations and heterologous prime-boost regimens, hold promise for improving immunogenicity in profoundly immunosuppressed patients [25]. The development of point-of-care assays to rapidly assess immune responses could facilitate real-time clinical decision-making regarding booster doses.

Future research should focus on several key areas to advance vaccination practices in oncology patients. First, identifying reliable biomarkers of vaccine responsiveness will enable truly personalized vaccination strategies [59,60]. This includes not only measuring antibody titers but also assessing T cell responses, B cell memory, and innate immune activation [16,17].

Second, optimizing the timing, type, and schedule of vaccines—including booster and heterologous regimens—can enhance both humoral and cellular immunity, particularly in patients receiving immunosuppressive therapies [38,39]. Third, novel adjuvants and vaccine platforms may overcome the limitations of current formulations in immunocompromised hosts [25]. Fourth, integration of vaccination into routine oncology care pathways, supported by multidisciplinary teams and standardized protocols, will be essential for improving uptake [53,59,61].

Finally, the development of universal vaccines—such as universal influenza vaccines targeting conserved hemagglutinin stalk antigens and pan-coronavirus vaccines designed to elicit cross-reactive immunity—holds particular promise for immunocompromised populations who face challenges from repeated vaccination and suboptimal responses to current formulations. These next-generation platforms could reduce the burden of annual revaccination and provide broader, more durable protection in oncology patients [25].

## 7. Conclusions

Vaccination is an essential component of comprehensive oncology care. Despite reduced immune responses in some patient subgroups, the clinical benefits of vaccination clearly outweigh potential limitations. Current evidence supports the safety and effectiveness of inactivated vaccines across diverse oncology populations, including those receiving immune checkpoint inhibitors, targeted therapies, and conventional chemotherapy.

Personalized strategies, booster dosing, and integrated care models are crucial to maximizing vaccine effectiveness in cancer patients. Overcoming organizational barriers and addressing vaccine hesitancy through education and systematic implementation strategies remain as priorities for improving vaccination coverage. As new vaccine technologies emerge and our understanding of immune responses in cancer patients deepens, vaccination strategies will continue to evolve to provide optimal protection for this vulnerable population.

## Figures and Tables

**Table 1:** Summary of vaccine evidence in oncology patients.

Vaccine	Recommendation	Immunogenicity	Safety Profile	Special Consideration	Refs.
Influenza (inactivated)	Universally recommended annually	Solid tumors: 52–84% seroconversion Hematological malignancies: reduced but still beneficial Safe during ICIs therapy	Excellent safety profile. No increased immune-related adverse events with ICIs	High-dose vaccine preferred in HSCT recipients Adjuvanted vaccines may improve responses Can be given during chemotherapy	[19,20,21,23]
Pneumococcal conjugate (PCV13/PCV15/PCV20)	Recommended for all cancer patients	>64% for most serotypes Superior to polysaccharide vaccines in immunocompromised hosts T cell-dependent response	Well tolerated. Low rate of adverse events	Sequential strategy: PCV followed by PPSV23 Associated with decreased pneumonia-related hospitalizations Preferably before immunosuppressive therapy	[25,26,27,28,31]
SARS-CoV-2 (mRNA)	Recommended (primary series + boosters)	Solid tumors: >90% seroconversion Hematological malignancies: 40–70% seroconversion B cell depletion therapy: markedly reduced T cell responses often preserved	Safe and well tolerated. No interference with cancer therapy	Additional booster doses significantly improve responses Consider immune monitoring in high- risk patients Cellular immunity may compensate for low antibody titers	[17,32,33,34,35,36,37,38,44]
Herpes Zoster (recombinant, RZV)	Recommended for adults ≥ 50 years and immunocompromised adults ≥ 19 years	Hematological malignancies: >60% seroconversion Vaccine efficacy: >68% in immunosuppressed Strong cellular and humoral responses Excellent response during ICI therapy	Very good safety profile. Superior to live vaccine. No risk of vaccine-associated zoster	Preferred over live zoster vaccine Two-dose series (0, 2–6 months) AS01B adjuvant enhances immunity Can be given during most cancer therapies	[40,41,42,43,45,46]
Hepatitis B (recombinant)	Recommended for at-risk patients, especially before B cell-depleting therapy or HSCT	Variable responses depending on immunosuppression level	Well tolerated	Essential to prevent viral reactivation Test for immunity before starting immunosuppressive therapy May require higher doses or additional doses Consider adjuvanted formulations	[47]
HPV (recombinant)	Recommended for eligible age groups and young cancer survivors	Standard responses in most patients when given before/after cancer therapy	Excellent safety profile	Important for primary and secondary cancer prevention Young cancer survivors at higher risk of HPV-related secondary malignancies Preferably before immunosuppressive therapy	[48]

## Data Availability

Not applicable.

## Abbreviations

The following abbreviations are used in this manuscript:

ACIP	Advisory Committee on Immunization Practices
AS01B	Adjuvant System 01B
ASCO	American Society of Clinical Oncology
BTK	Bruton’s tyrosine kinase
CD20	Cluster of Differentiation 20
CD38	Cluster of Differentiation 38
COVID-19	Coronavirus Disease 2019
EHR	Electronic health record
ESMO	European Society for Medical Oncology
HCV	Healthcare worker
HPV	Human Papillomavirus
HSCT	Hematopoietic Stem Cell Transplantation
ICI	Immune Checkpoint Inhibitor
IDSA	Infectious Diseases Society of America
IPD	Invasive Pneumococcal Disease
MF59	Oil-in-water emulsion adjuvant
mRNA	Messenger Ribonucleic Acid
PCP	Primary care physician
PCV13	13-valent Pneumococcal Conjugate Vaccine
PCV15	15-valent Pneumococcal Conjugate Vaccine
PCV20	20-valent Pneumococcal Conjugate Vaccine
PPSV23	23-valent Pneumococcal Polysaccharide Vaccine
RZV	Recombinant Zoster Vaccine (Shingrix)
SARS-CoV-2	Severe Acute Respiratory Syndrome Coronavirus 2
VPD	Vaccine-Preventable Disease
